# Coronary Obstruction
*A Paper read to the Bath and Bristol Branch of the British Medical Association, October 26th, 1927.


**Published:** 1927

**Authors:** Carey F. Coombs

**Affiliations:** Physician to the Bristol General Hospital


### CORONARY OBSTRUCTION.*

BY

CAREY F. COOMBS, M.D., F.R.C.P. Lond.,
*Physician to the Bristol General Hospital.*

---

SUDDEN occlusion of a coronary artery is a grave accident, which in some instances at least has been proved at autopsy to lead to gradual or rapid necrosis of the myocardium. The diagnosis of the condition during life is not easy, but I venture to suggest that it gives rise to a definite and recognisable syndrome.

Rather over twenty years ago I was attending an old clergyman with a high blood pressure and thick arteries for a stroke of the thrombotic type, when he had a severe attack of substernal pain, followed by the development of an acute pericarditis. The obvious interpretation of these facts was that the same thing had happened in his coronary circulation as that which had already occurred within the arteries of his brain; in both an artery had become obstructed, and the area supplied had undergone some degree of necrosis. However, he recovered, and I had no other experiences of the kind for some time, except that two or three years later a gentleman of 60 odd, to whom I was called as he was dying in the street after what appeared to be a severe anginal attack, proved to have a rent in the wall of his right ventricle which apparently arose out of a similar necrotic process. This, again, recalled the case of a man admitted to St. Mary's Hospital

* A Paper read to the Bath and Bristol Branch of the British Medical Association, October 26th, 1927.

while I was a student. He also was elderly, and after a very bad bout of anginal pain—I can still recollect the pallor of his face and his expression of extreme suffering—developed a friction rub over his heart. The area of this increased rapidly, and he died in four or five days, the autopsy revealing in his case also a tear about an inch long in the wall of the right ventricle.

When in 1911 Hochhaus[1] and in 1912 Herrick[2] published papers describing the clinical picture displayed by patients in whom sudden coronary obstruction was found to have occurred, it was clear that my surmise about the original patient had been correct; but although I have since that time made a diagnosis of coronary obstruction in 36 patients, more than half of whom have died within the year, it is a most curious fact that there has not been a single post-mortem examination. Nevertheless, I feel sure that the diagnosis is well founded, for many papers have since been published, in this country[3] and elsewhere, but more especially in America, proving that there is a real and constant relation between the syndrome that I am about to describe and sudden or rapid occlusion of a coronary artery.

In all the 36 patients to whom these observations refer the first phase of the attack has been a paroxysm of substernal pain. Usually it is behind the manubrium, occasionally beneath the xiphisternum. When its centre is epigastric the pain may be misinterpreted as gallstone colic, or as some form of acute dyspepsia. From this central origin it radiates into the arms, the neck, even into the lower jaw, showing in all these situations a predilection for the left side. Occasionally, also, it is felt between the shoulders. It is usually agonising and terrifying, having in it an element of that sense of oppression which made "peine forte et dure"

so inhuman a punishment. In 17 instances the patients had had no previous experience of similar pain, and in 8 others only during the previous few days or weeks. Usually the attack begins without obvious provocation ; often, indeed, it came on while the patient was in bed at night.

Two things strike one as soon as one sees the patient : his expression of intense suffering and his pallor. I find that some such adjective as "pallid," "ashen," "livid" appears in my notes of all but two cases. The picture is not easily forgotten. The patient lies motionless, his face quite colourless and bedewed with sweat, his expression that of one who is either enduring torment or anticipating a repetition of it. In 11 cases there was vomiting in this early stage, and in two hæmoptysis, facts which served to obscure rather than to enlighten the diagnosis.

But it is the subsequent evolution of the attack which makes diagnosis possible. In several patients within a few hours after the onset, more often perhaps after an interval of one or two days, there were added to the initial pain and collapse evidences of gross injury to the cardiac wall. The most characteristic picture displays signs not only of serious myocardial disease, but also of pericardial inflammation. In 16 of my 36 patients friction was noted over the præcordium, usually beginning a day or two after the onset of symptoms and disappearing inside a week. Some direct evidence of a grave myocardial lesion was manifest in nearly every case. The function which appeared to suffer most often was the contractile power of the left ventricle. The heart sounds were noted to be weak in 25 instances. This enfeeblement develops rapidly and may be most striking, the sounds becoming quite inaudible in some cases. If the attack runs a favourable course, the sounds recover their strength in the space of two or three days.

Beside mere weakening, the sounds may exhibit other changes indicative of an extreme degree of contractile failure. A triple cantering rhythm was heard in 10 of my cases ; in several embryocardia was noted ; and in 5 a systolic murmur developed at the apex. Two other signs of ill omen in that they indicate a failure of ventricular force are detected by the use of the sphygmomanometer. In 16 of my patients the pulse was alternating at some stage of the attack, the degree of alternation being so slight in most instances as to escape detection except under the test of auscultation over the brachial artery distal to an inflated armlet. Even more ominous, perhaps, is a sharp fall in the blood pressure. This was noted in 17 of my 36 patients. What is most characteristic is a fall in the systolic pressure out of proportion to the fall of diastolic pressure. For example, in one patient the reading at the beginning of the attack was 130 mm. Hg. systolic, 80 diastolic, while a few weeks later the systolic pressure had fallen to 100, while the diastolic figure remained at 80. Even where comparison with an earlier measurement is not possible, the discovery of an abnormally narrow pulse pressure is highly significant. Another sign of acute ventricular distress is a rapid increase in the area of the cardiac dulness, similar to that which is sometimes seen in diphtheria and in acute rheumatic carditis. In one instance examination of the patient six or seven weeks after the initial pain disclosed an area of heaving pulsation in the first, second and third left intercostal spaces extending from the sternal border as far as the anterior border of the axilla. In the same area a diastolic shock was felt and the percussion note was dull. There was little doubt in my mind that these signs pointed to the development of an aneurysm of the cardiac wall, but unfortunately no autopsy was possible when the patient died six months later.

Other interesting and significant proofs of myocardial damage were noted in some of my patients. Five of them developed the total arrhythmia characteristic of auricular fibrillation. In two there was a bradycardia that was almost certainly indicative of auriculo-ventricular block, and in another a speeding that suggested the onset of auricular flutter. In none of these patients was it possible to apply graphic methods at the time of the disturbance. Unfortunately, it was impossible to subject most of these patients to electro-cardiographic examination, and I have therefore no evidence of this kind to offer you. Neither have I made systematic blood counts, but those who have done so find that the polymorphonuclear leucocytes are appreciably increased in many cases. Libman[4] has shown that this leucocytosis may persist after other signs of actual myocardial destruction have died away. Slight fever is by no means uncommon; it was noted in 8 of my 36 patients, and might doubtless have been found in others if it had been possible to make a chart extending over the whole attack in every case.

In several patients the phase of convalescence has been complicated by the development of physical signs at the bases of the lungs that are suggestive of an imperfect consolidation. The course followed, however, has been that of massive collapse rather than of a pneumonic infection.

It is remarkable that it should ever be possible to recover from so calamitous an experience as this. Yet exactly one quarter of my patients have made a recovery which appears to stand the test of time. Of the 27 who did not survive, rather more than half succumbed without having recovered enough to get up and about again.

There are a great many points of interest that lend themselves to discussion. For instance, there is the fact that in these, which are to all intents and purposes attacks of angina with an anatomical basis, that basis is cardiac and not aortic. To my mind this piece of evidence goes far to settle the old controversy about the site of anginal pain in favour of the cardiac as against the aortic view. Another consideration of some practical importance is the possibility of foreseeing the liability of any given patient to catastrophes of this sort. If there had been time I should have liked to offer you some account of mistakes made over the diagnosis of cardiac pain, mistakes which have been rudely corrected when patients who had been thought to owe their attacks of pain to some source that was not the heart have died suddenly, or at all events under circumstances very strongly suggestive of coronary obstruction. Three or four of these people have been young or in early middle life, and have shown no sign of cardiac disease. Conversely, I have known a patient who had recovered from an attack of coronary obstruction to have subsequent attacks of cardiac pain without evidence of added injury to the heart muscle.

There is only time to discuss briefly one other side of this topic, that of causation. Of my 36 patients 29 were men, 7 only being women. In 29 instances the type of heart disease present was labelled by me as senile, and in 4 as hyperpietic. By a curious coincidence the first and the last patients in this series were father and son, the latter presenting, at the age of 45, a remarkably thick and hard brachial artery and a systolic blood pressure of 210. There were only two other patients under the age of 50, and in both of these there was a hint of an infective element in the obstruction, for one was the subject of a chronic *B. coli* cystitis, the other of an acute erysipelas. Six of them were between 50 and 60, seventeen between 60 and 70, six of them between 70 and 80, and three of them over 80. I was surprised to find so striking a testimony to the importance of age as a predisposing factor. Tested from the opposite point of view, this fact still holds good. Nearly 15 per cent. of a series of cases of senile heart disease exhibited symptoms of sudden or acute coronary obstruction, of patients with myocardial degeneration associated with high blood pressure less than 3 per cent., while of those with cardiac syphilis not a single one betrayed any sign of this complication. The preponderating value of senile degeneration as a causal factor is strongly in favour of the view usually held that regards these obstructions as due to thrombosis. The improbability of an embolic origin is attested by the fact that I have seen no example of this syndrome among patients suffering from ulcerative endocarditis or from rheumatic heart disease.

In four patients other thromboses had occurred at or near about the same time, thrice in the veins of the lower limbs and once in the arteries of the mesentery and left arm. Finally, there was the case of a man of 49 who struggled through a most alarming attack which developed during the decline of a very severe facial erysipelas.

I should have liked to include a discussion of other types of coronary obstruction. There are those in whom death occurs so rapidly as to preclude an accurate diagnosis. In others, again, the obstruction develops so slowly that it is not accompanied by pain or other striking symptoms, and it is only disclosed later at an autopsy. As this paper is purely clinical, it takes no cognisance of such facts as these, except to remark that my experience has been true to type, since it has included examples of failure to diagnose both of these kinds of obstruction.

REFERENCES.

[1] Hochhaus, *Deut. Med. Woch.*, 1911, 2065.
[2] Herrick, *Jour. Amer. Med. Assoc.*, 1912, i. 1971.
[3] Gibson, *Lancet*, 1925, ii. 1270.
[4] Libman, *Amer. Heart Jour.*, 1927, ii. 321.

